# SYN-007, an Orally Administered Beta-Lactamase Enzyme, Protects the Gut Microbiome from Oral Amoxicillin/Clavulanate without Adversely Affecting Antibiotic Systemic Absorption in Dogs

**DOI:** 10.3390/microorganisms8020152

**Published:** 2020-01-22

**Authors:** Sheila Connelly, Brian Fanelli, Nur A. Hasan, Rita R. Colwell, Michael Kaleko

**Affiliations:** 1 Synthetic Biologics, Inc., Rockville, MD 20850, USA; mkaleko@syntheticbiologics.com; 2 CosmosID, Inc., Rockville, MD 20850, USA; brian.fanelli@cosmosid.com (B.F.); nur.hasan@cosmosid.com (N.A.H.); rita.colwell@cosmosid.com (R.R.C.); 3 College of Computer, Mathematical, & Natural Sciences, University of Maryland Institute of Advanced Computer Studies, College Park, MD 20742, USA

**Keywords:** antibiotic, gut microbiome, beta-lactam, beta-lactamase, beta-lactamase inhibitor

## Abstract

Beta-lactamases, enzymes produced by bacteria to degrade beta-lactam antibiotics, have been harnessed as therapeutics to protect the gut microbiome from damage caused by antibiotics. Proof-of-concept of this approach using SYN-004 (ribaxamase), a beta-lactamase formulated for oral delivery with intravenous (IV) penicillins and cephalosporins, was demonstrated with animal models and in humans. Ribaxamase degraded ceftriaxone in the gastrointestinal tract, protected the gut microbiome, significantly reduced the incidence of *Clostridioides difficile* disease and attenuated emergence of antibiotic resistant organisms. SYN-007 is a delayed release formulation of ribaxamase intended for use with oral beta-lactams. In dogs treated with oral amoxicillin, SYN-007 diminished antibiotic-mediated microbiome disruption and reduced the emergence of antibiotic resistance without altering amoxicillin systemic absorption. Here, SYN-007 function in the presence of clavulanate, a beta-lactamase inhibitor, was investigated. Dogs received amoxicillin (40 mg/kg, orally (PO), three times a day (TID)) or the combined antibiotic/beta-lactamase inhibitor, amoxicillin/clavulanate (40 mg/kg amoxicillin, 5.7 mg/kg clavulanate, PO, TID) +/™ SYN-007 (10 mg, PO, TID) for five days. Serum amoxicillin levels were not significantly different +/™ SYN-007 compared to amoxicillin alone or amoxicillin/clavulanate alone as controls for both first and last doses, indicating SYN-007 did not interfere with systemic absorption of the antibiotic. Whole genome shotgun metagenomics analyses of the fecal microbiomes demonstrated both amoxicillin and amoxicillin/clavulanate significantly reduced diversity and increased the frequency of antibiotic resistance genes. Microbiome damage appeared more severe with amoxicillin/clavulanate. In contrast, with SYN-007, microbiome diversity was not significantly altered, and frequency of antibiotic resistance genes did not increase. Importantly, SYN-007 functioned in the presence of clavulanate to protect the gut microbiome indicating that SYN-007 activity was not inhibited by clavulanate in the dog gastrointestinal tract. SYN-007 has the potential to expand microbiome protection to beta-lactam/beta-lactamase inhibitor combinations delivered orally or systemically.

## 1. Introduction

The gut microbiome, composed of the commensal microbiota and their genetic material, represents a complex ecosystem important to human health. Antibiotics disrupt this bionetwork, resulting in alterations of the normal microbial balance that can weaken colonization resistance and result in pathobiont overgrowth. Broad-spectrum antimicrobials, of which the beta-lactams are the most commonly used, are especially harmful to the beneficial anaerobes inhabiting the gut [1,2,3], and have been associated with increased risk of *Clostridioides difficile* infection (CDI) [4,5]. In addition, the gut microbiome functions as a reservoir of antibiotic resistance [6]. Selective pressure caused by antimicrobial use promotes the emergence and evolution of pathogens by accelerating the transfer of antibiotic resistance genes [6,7]. Therefore, limiting the exposure of the gut microbiota to antimicrobials by their inactivation in the gastrointestinal (GI) tract is a strategy to preserve the gut microbiome and reduce antibiotic resistance.

Beta-lactamases are enzymes naturally produced by bacteria that specifically inactivate beta-lactam antibiotics via hydrolysis. SYN-004 (ribaxamase) is a beta-lactamase enzyme formulated for oral administration and intended for use with intravenous (IV) beta-lactams to degrade antibiotics excreted through bile into the GI tract to protect the intestinal microbiota. Ribaxamase is formulated with a pH-sensitive enteric coating that protects the beta-lactamase from stomach acid and proteases and releases the enzyme at pH 5.5 or greater, the pH of the upper small intestine, proximal to the site of bile release [8]. In animals and humans, ribaxamase was demonstrated to degrade IV administered ceftriaxone in intestinal fluid, preserve the gut microbiome, and attenuate antibiotic resistance [9,10,11]. In a Phase 2b clinical study of more than 400 patients, ribaxamase significantly reduced the incidence of CDI in hospitalized patients receiving IV ceftriaxone, without interfering with antibiotic efficacy in treating the underlying infection and reduced antibiotic-mediated damage of the gut microbiome [12,13].

**Figure 1:**
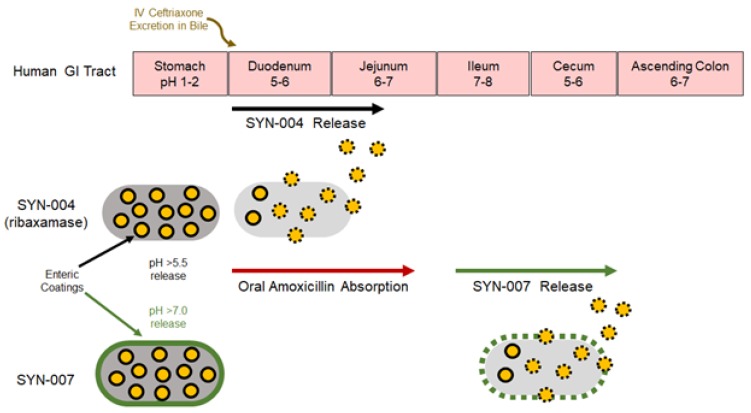
Schematic representation of SYN-007 intestinal dissolution profile. SYN-007 is composed of enteric-coated beta-lactamase pellets (ribaxamase) within an enteric-coated capsule. Following oral administration of SYN-007, the enteric coating of the capsule remains intact until the pH reaches >7.0 in the lower small intestine, where the capsule enteric coating dissolves releasing the enteric-coated ribaxamase pellets that rapidly dissolve at pH ≥ 5.5 to release the beta-lactamase enzyme in the small intestine, distal to the site of oral antibiotic systemic absorption [15]. In contrast, ribaxamase is composed of enteric-coated beta-lactamase pellets within an uncoated, hard capsule. After swallowing, ribaxamase pellets are released in the stomach, pass intact into the duodenum and rapidly dissolve at pH ≥ 5.5 releasing the beta-lactamase enzyme in the upper small intestine [17].

However, the majority of beta-lactams are delivered orally, not systemically [14], and the ribaxamase formulation is not appropriate for co-administration with oral beta-lactams [15]. Amoxicillin, and other oral beta-lactams, are absorbed from the proximal small intestine [16], the site of ribaxamase release [8]. Indeed, administration of ribaxamase with oral amoxicillin in dogs resulted in no detectable antibiotic in the blood, indicating that ribaxamase degraded the antibiotic within the GI tract prior to its systemic absorption [15]. A novel formulation of ribaxamase was developed for use with oral beta-lactams [15]. This delayed release formulation of ribaxamase, SYN-007, targets enzyme release to the lower small intestine distal to the site of oral beta-lactam absorption [15] (Figure 1). SYN-007 employs a dual coating approach using enteric-coated ribaxamase pellets [8] packaged into enteric-coated capsules [15]. The enteric coating of the capsule dissolves at pH > 7.0, the pH of the ileum in the lower small intestine [17]. After capsule dissolution, enteric-coated enzyme pellets rapidly release an active enzyme capable of degrading the antibiotic prior to its reaching and harming the colonic microbiota [15].

The efficacy of SYN-007 in protecting the gut microbiome was evaluated in dogs [15]. Amoxicillin blood levels in animals that were co-administered oral SYN-007 and oral amoxicillin were not significantly different from those of animals that received amoxicillin alone, demonstrating that the beta-lactamase enzyme was not released prematurely in the canine GI tract [15]. In animals that received SYN-007, gut microbiome composition and diversity were maintained, and the emergence of antibiotic resistance genes was attenuated [15]. These data demonstrate that SYN-007 protected the gut microbiome from antibiotic-mediated damage without interfering with oral amoxicillin systemic absorption [15].

Broadening microbiome protection to include oral beta-lactam antibiotics greatly expands the utility of oral beta-lactamase therapy. Oral amoxicillin is the most commonly used antimicrobial, and alone or in combination with the beta-lactamase inhibitor, clavulanate, accounted for over 70 million prescriptions in the U.S. in 2016 [14]. Amoxicillin/clavulanate, approved in the U.S. in 1984, is used to treat resistant infections caused by beta-lactamase-producing pathogens, and is routinely administered to patients who fail therapy with amoxicillin alone [18]. However, amoxicillin/clavulanate incurs more adverse side effects than amoxicillin alone, including increased incidence of antibiotic-associated diarrhea (AAD), which can be severe [18]. AAD is associated with antibiotic-mediated disruption of the gut microbiome [18]. Therefore, protection of the gut microbiome from amoxicillin/clavulanate remains an unmet need. However, SYN-007 function in the presence of a beta-lactamase inhibitor had not been demonstrated.

Here, SYN-007 efficacy was evaluated in dogs that received oral amoxicillin or oral amoxicillin/clavulanate with or without SYN-007 for five days. After the first and last doses, serum antibiotic levels were measured to evaluate whether SYN-007 interfered with amoxicillin systemic absorption. Metagenomic and resistome analyses of fecal DNA that was collected before and after antibiotic exposure was performed to assess changes to the gut microbiome and gut resistome.

## 2. Materials and Methods

### 2.1. Test Article

SYN-007 comprises of sugar pellets coated with ribaxamase and then covered with Eudragit^®^ L30-D55 (Evonik, Essen, Germany) [8]. The prepared enzyme pellets were loaded into size 9h capsules (2.69 mm diameter by 5.1 mm length) that were banded and covered with Eudragit^®^ FS30D (Evonik) by spray coating as previously described [15]. A 10 mg dose of SYN-007 was composed of eight filled and coated 9h capsules contained within one size 0 uncoated hard capsule [15]. SYN-007 was manufactured and tested by Aptuit, an Evotec Company, Greenwich, CT, USA (formerly Kuecept, Ltd.) [15].

### 2.2. Animals and Test Article Administration

Female Beagle dogs, 7 to 8 months old and 6.5 to 7.7 kg, were purchased from Covance Research Products (Denver, PA, USA). Animals that were never exposed to antibiotics were ordered specifically for this study. Animals were received at the test site, Calvert Laboratories, Inc. (Scott Township, PA, USA), 30 days before study initiation and first sample collection to allow acclimation to their new environment. Animal health was evaluated daily. Individual animal housing was in compliance with United States Department of Agriculture Guidelines and animals were allowed to comingle prior to study initiation. Animals received antibiotic-free PMI Canine Diet. Animals were randomly divided into 4 cohorts (*n* = 5 each); Amoxicillin alone, Amoxicillin + SYN-007, Amoxicillin/Clavulanate alone, and Amoxicillin/Clavulanate + SYN-007.

On study days 1–5, dogs received food three times per day, 1.5 hours (h) after each antibiotic +/™ SYN-007 dose. On study day 6, animals were fed 1.5 h after the last dose of antibiotic +/™ SYN-007. Body weights, recorded daily, were used to calculate the dose of amoxicillin or amoxicillin/clavulanate received on study days 1–6 to achieve 40 mg/kg per dose. Dogs were allowed free access to water.

Amoxicillin was supplied as a powder (NDC 0781-6157-52, McKesson Corporation, Irving, TX, USA) and resuspended in 100% Mott’s Apple Juice (pH 3.0) instead of water, per package instructions, at 400 mg/5 mL as previously described [15]. Augmentin (amoxicillin and clavulanate potassium) was supplied as a powder (NDC 0143-9982-01, West-Ward Pharmaceuticals, Eatontown, NJ, USA) and resuspended in 100% Mott’s Apple Juice (pH 3.0) instead of water, per package instructions, at 400 mg amoxicillin + 57 mg clavulanate potassium/5 mL. Animals received amoxicillin (40 mg/kg) or amoxicillin/clavulanate (40 mg/kg amoxicillin + 5.7 mg/kg clavulanate) three times a day at 8 h intervals, with or without one capsule of SYN-007 (10 mg), with the last dose on the morning of day 6, for a total of 16 doses of antibiotic +/™ SYN-007 as previously described [15]. Animals received an additional 5 mL Mott’s apple juice delivered orally with a 10 mL plastic syringe after each antibiotic dose. After the antibiotic was delivered to all animals, the appropriate dogs received one capsule of SYN-007 orally followed by another 5 mL of apple juice to ensure that the SYN-007 capsule was swallowed as previously described [15]. Blood was collected after the first dose on day 1 and the last dose on day 6 at 0.5, 1, 2, 3, 4, 6, and 8 h after antibiotic administration, and serum collected as previously described [15]. Fecal samples were collected twice, on prior to antibiotic dosing on day ™1 and on day 6 following antibiotic +/™ SYN-007 dosing as previously described [15].

The animal study protocol, 0832DS123.002, was approved on 8 February 2018 by Calvert Laboratories, Inc., Institutional Animal Care and Use Committee (IACUC). All animal procedures were performed in accordance with the Animal Welfare Act at Calvert Laboratories, Inc. (Scott Township, PA, USA) and in agreement with the guidelines established by the Calvert Institutional IACUC. Calvert Laboratories, Inc. is fully accredited by the Association for Assessment and Accreditation of Laboratory Animal Care (AALAC).

### 2.3. Amoxicillin Serum Measurement

Liquid chromatography turbo ion spray tandem mass spectrometry (LC/MS/MS) was used for amoxicillin serum measurements as previously described [15]. An amoxicillin serum analysis assay was developed and performed by Sannova Analytical, Inc. (Somerset, NJ, USA). GraphPad Prism 7 (GraphPad Software, San Diego, CA, USA) was used to calculate area under the curve and for statistical analyses.

### 2.4. Fecal DNA Extraction, Whole Genome Shotgun Sequencing, and Metagenomic Analyses

Total DNA was isolated from fecal specimens, and libraries were constructed, quantified, and sequenced using a single Illumina HiSeq v3 flowcell aiming for 25 million 100 bp reads per sample as prevoiusly described [15]. Open source BBDuk software from BBTools (https://jgi.doe.gov/data-and-tools/) was used to evaluate read quality prior to metagenomics analyses. Samples had an average read quality of Q20 demonstrating 99% sequencing accuracy (https://www.illumina.com/science/education/sequencing-quality-scores.html). Reads per sample were 60,000,000 ± 9,000,000 (mean and standard deviation), demonstrating similar read depth.

CosmosID, Inc. (Rockville, MD, USA) bioinformatics software was used to analyze unassembled whole genome metagenomic sequencing, as previously described [15], for bacterial identification to species and subspecies levels for relative abundance quantifications. Resistome analysis was performed to identify antibiotic resistance genes and their frequency, as previously described [15].

Bacterial metagenomics data were analyzed for alpha diversity using the Shannon Index [19], and beta-diversity using principal component analyses. The NMF R software package [20] was employed to generate stacked bar graphs based on microorganism relative abundance. Antibiotic-resistance genes were identified based on percentage of gene coverage as a function of gene-specific read frequency as previously described [15]. Microsoft Excel 2016 (Microsoft Corporation, Redmond, WA, USA) or GraphPad Prism 7 (GraphPad Software, San Diego, CA, USA) were used for statistical analyses.

### 2.5. Data Availability

Fecal DNA metagenomics sequencing data are available in Sequence Read Archive (SRA) (https://submit.ncbi.nlm.nih.gov/subs/sra/), Accession SRP093227.

## 3. Results

### 3.1. SYN-007 did not Significantly Affect Oral Amoxicillin Systemic Absorption

**Figure 2:**
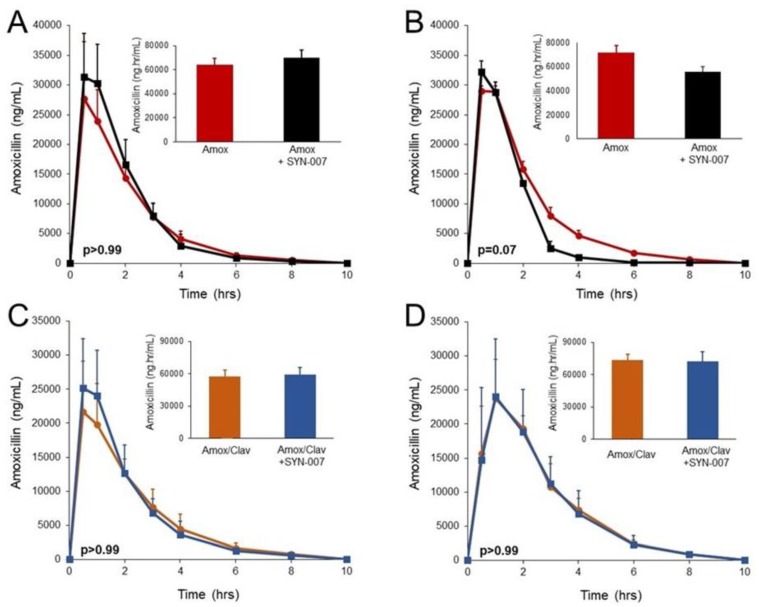
Amoxicillin serum levels. Amoxicillin was measured in dog serum collected at the indicated times. Inset bar graphs display the area under the curve (AUC) for each group. (**A**) Amoxicillin levels after the first dose of amoxicillin (Day 1) +/™ SYN-007. (**B**) Amoxicillin levels after the last (16^th^) dose of amoxicillin (Day 6) +/™ SYN-007. Black: Amoxicillin alone; red: Amoxicillin + SYN-007. (**C**) Amoxicillin levels after the first dose of amoxicillin/clavulanate (Day 1) +/™ SYN-007. (**D**) Amoxicillin levels after the last (16^th^) dose of amoxicillin (Day 6) +/™ SYN-007. Orange: Amoxicillin/clavulanate alone; blue: Amoxicillin/clavulanate + SYN-007. Data are displayed as mean + standard deviation (*n* = 5). *p* values were obtained by analysis of the AUC from each group for each collection day using Kruskal-Wallis non-parametric ANOVA with Dunn’s multiple comparisons test (Graphpad Prism 7) by comparing the AUC from each SYN-007 group to the appropriate antibiotic alone group, amoxicillin alone or amoxicillin/clavulanate alone. *p* values for each comparison are displayed.

SYN-007, a delayed release formulation of ribaxamase, composed of enteric-coated ribaxamase pellets [8] within an enteric-coated capsule (Figure 1) [15], was tested in dogs that received oral amoxicillin or oral amoxicillin/clavulanate. Animals received an oral antibiotic three times a day for five days with their final dose on day 6. The appropriate groups of dogs also received oral SYN-007 immediately following antibiotic delivery. Serum, collected from each dog after the first antibiotic dose +/™ SYN-007 on day 1 and the last dose on day 6, was evaluated for amoxicillin concentrations, and the area under the curve (AUC) for each SYN-007 cohort was compared to that of the appropriate amoxicillin or amoxicillin/clavulanate alone control group (Figure 2). No significant difference in amoxicillin serum level AUC for any cohort was observed on day 1 (Figure 2A,C; *p* > 0.99). Similarly, by day 6, after 16 doses of antibiotic + SYN-007, there was no significant difference in amoxicillin serum level AUC for either group (Figure 2B,D; *p* = 0.07 and *p* > 0.99, respectively). Notably, amoxicillin blood levels appeared to be lower at the 3, 4, and 6 h time points in the amoxicillin + SYN-007 cohort, although this difference was not significant (Figure 2B). In contrast, following 16 doses of amoxicillin/clavulanate + SYN-007, the amoxicillin serum level curves were nearly identical and superimposable (Figure 2D). These data indicate that SYN-007 did not significantly interfere with oral amoxicillin systemic absorption.

### 3.2. SYN-007 Reduced Oral Amoxicillin-Mediated Microbiome Damage

**Figure 3:**
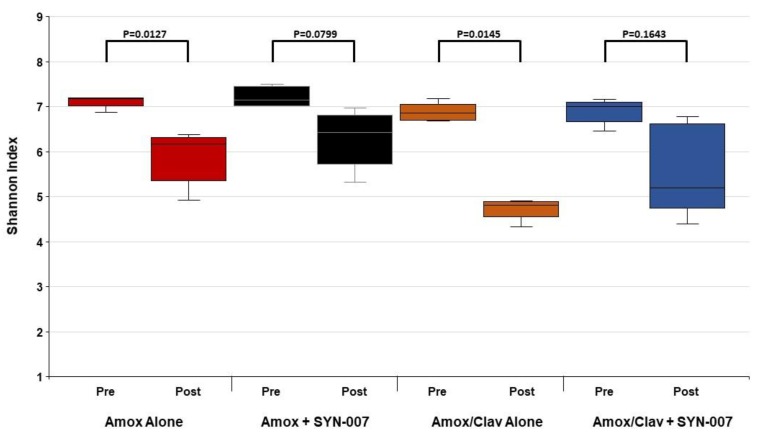
Dog fecal microbiome alpha diversity before and after antibiotic exposure. Shannon indices calculated using the fecal microbiome metagenomics data are displayed for each cohort (*n* = 5) as box plots. *p* values were obtained using Kruskal-Wallis non-parametric ANOVA with Dunn’s multiple comparisons test (Graphpad Prism 7) by comparing pretreatment Shannon indices (Pre) to post-treatment Shannon indices (Post). Red: Amoxicillin alone; black: Amoxicillin + SYN-007; orange: Amoxicillin/Clavulanate alone; blue: Amoxicillin/Clavulanate + SYN-007.

Changes to the gut microbiome were assessed by determining the relative abundance of each microorganism in each fecal sample followed by microbiome composition comparisons. Calculation of alpha diversity using the Shannon index revealed that amoxicillin or amoxicillin/clavulanate alone resulted in significantly lower Shannon indexes, compared to pretreatment (*p* = 0.0127 and 0.0145, respectively; Figure 3). In contrast, pre- and post-treatment Shannon indices were not significantly different for amoxicillin + SYN-007 and amoxicillin/clavulanate + SYN-007 cohorts (*p* = 0.0799 and 0.1643, respectively; Figure 3). These data indicate that SYN-007 protected the gut microbiome from oral antibiotic-mediated damage in the absence and presence of the beta-lactamase inhibitor, clavulanate.

**Figure 4:**
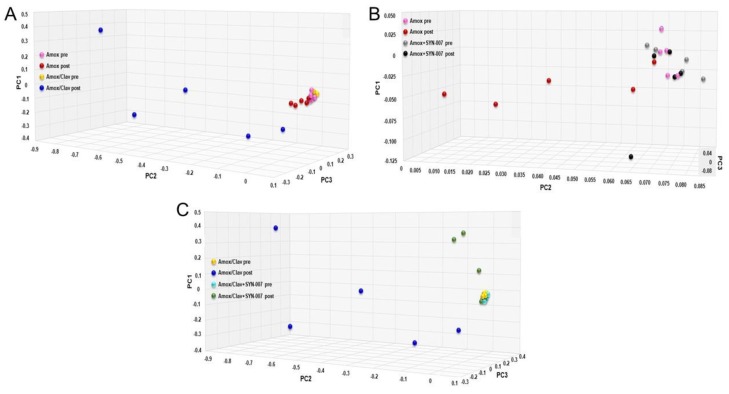
Principal coordinate analyses of fecal microbiomes. Jaccard dissimilarity was used to compare fecal microbiomes [21]. (**A**) Amoxicillin alone vs. Amoxicillin/Clavulanate alone. Pink: Amoxicillin pretreatment; red: Amoxicillin post treatment; yellow: Amoxicillin/clavulanate pretreatment; dark blue: Amoxicillin/clavulanate post-treatment. (**B**) Amoxicillin alone vs. Amoxicillin + SYN-007. Pink: Amoxicillin pretreatment; red: Amoxicillin post-treatment; gray: Amoxicillin + SYN-007 pretreatment; black: Amoxicillin + SYN-007 post-treatment. (**C**) Amoxicillin/Clavulanate alone vs. Amoxicillin/Clavulanate + SYN-007. Yellow: Amoxicillin/clavulanate pretreatment; dark blue: Amoxicillin/clavulanate post-treatment; light blue: Amoxicillin/clavulanate + SYN-007 pretreatment; green: Amoxicillin/clavulanate + SYN-007 post-treatment.

Principle coordinate analysis (PCoA) comparing pretreatment microbiome composition to post-treatment was performed with a greater distance between points designating a more pronounced difference in microbiome composition (Figure 4). For ease of visualization, chosen cohorts were evaluated in separate analyses; amoxicillin alone vs. amoxicillin/clavulanate alone (Figure 4A), amoxicillin alone vs. amoxicillin + SYN-007 (Figure 4B), and amoxicillin/clavulanate alone vs. amoxicillin/clavulanate + SYN-007 (Figure 4C). All samples were included in a separate analysis (Supplemental Figure S1). Comparison of microbiomes exposed to the antibiotics alone revealed that pretreatment and amoxicillin post-treatment samples clustered more closely than amoxicillin/clavulanate post-treatment samples, indicating that microbiome composition was more disrupted by the antibiotic/beta-lactamase inhibitor combination than by antibiotic alone (Figure 4A). In the presence of SYN-007, pretreatment and antibiotic + SYN-007 samples clustered more closely than antibiotic alone post-treatment samples (Figure 4B,C). With amoxicillin +/™ SYN-007, pretreatment and amoxicillin + SYN-007 samples were not clustered as tightly, however, post-treatment amoxicillin alone samples appeared the most scattered (Figure 4B). In contrast, amoxicillin/clavulanate +/™ SYN-007 pretreatment samples clustered tightly, with amoxicillin/clavulanate post-treatment samples diverging the most from pretreatment (Figure 4C). Notably, 3 of 5 amoxicillin/clavulanate + SYN-007 post-treatment samples diverged from the pretreatment cluster compared to only one amoxicillin + SYN-007 sample (Figure 4B,C).

**Figure 5:**
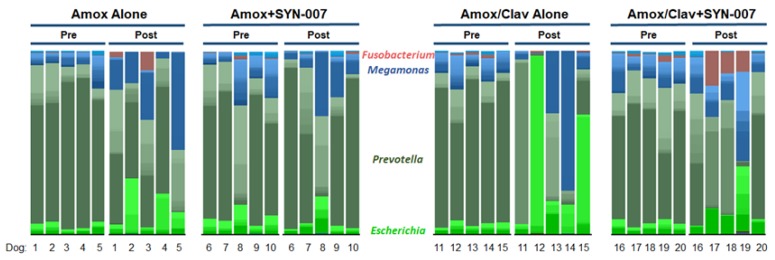
Stacked bar graph of fecal microbiomes (species level). Individual animal fecal microbiomes, pre- and post-antibiotic exposure, are displayed. The genera of selected species are displayed in the center, animal numbers are displayed on the bottom, and treatment groups and collection time point displayed at the top.

Stacked bar graphs, constructed based on the relative abundance of each bacterial species in each sample, were used to visualize specific changes in microbiota composition of the fecal microbiomes before and after antibiotic exposure (Figure 5). Compared to pretreatment microbiomes, amoxicillin or amoxicillin/clavulanate alone resulted in alterations of microbiome composition that were diminished in the presence of SYN-007 (Figure 5). Notably, overgrowth of *Escherichia coli* was observed following amoxicillin/clavulanate alone exposure in two dogs resulting in *E. coli* monodomination, defined as >30% of microbiome composed of one species (Dog 12, 92% and Dog 15, 56%) [22]. Likewise, two amoxicillin/clavulanate alone dogs displayed monodomination with *Megamonas hypermegale* (Dog 14, 55%) or *Bacteroides vulgantus* (Dog 11, 88%). Species monodomination was not observed in the amoxicillin alone, or either SYN-007 cohorts. However, increased relative abundance of *E. coli* and/or *M. hypermegale* was observed in all amoxicillin alone treated dogs while increased abundance of *Fusobacterium mortiferum* was observed in two amoxicillin alone treated dogs and three amoxicillin/clavulanate + SYN-007-treated dogs. These data demonstrate that amoxicillin/clavulanate altered gut microbiome composition more drastically than amoxicillin alone, and that antibiotic-mediated changes to the gut microbiome were attenuated with SYN-007.

### 3.3. SYN-007 Attenuated Antibiotic Resistance Gene Propagation

**Figure 6:**
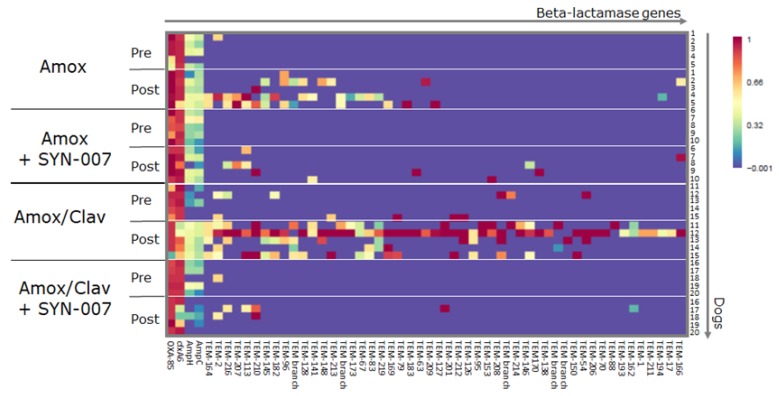
Frequency of beta-lactamase genes in dog fecal resistome. Heatmap analysis is displayed with rows representing individual animals at the indicated collection day (pre- or post-treatment). Resistance genes are identified at the bottom, treatment group and day of fecal collection on the left, and the animal numbers on the right. The color gradient key displays a linear scale of the percentage gene coverage as a measure of relative gene frequency.

Fecal resistomes were analyzed to evaluate if SYN-007 affected antibiotic resistance gene emergence and propagation. Heatmaps of antibiotic resistance genes in the fecal microbiome of each animal before and after antibiotic treatment were generated. Emergence of beta-lactamase genes, which confer resistance specifically to beta-lactam antibiotics, was observed following antibiotic exposure (Figure 6 and Supplemental Figure S2). The amoxicillin/clavulanate alone cohort displayed the most beta-lactamase genes post-treatment. Antibiotic alone cohorts displayed more beta-lactamase genes following antibiotic exposure than both SYN-007 cohorts post-treatment (Figure 6). The beta-lactamase genes that emerged were mainly those encoding class A TEM enzymes.

**Figure 7:**
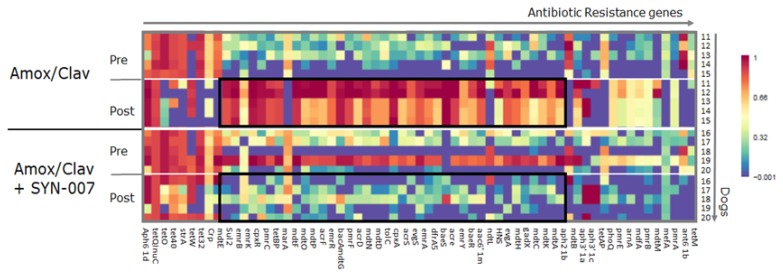
Frequency of antibiotic resistance genes, other than beta-lactamase genes, in dog fecal resistome. Heatmap analysis is displayed with rows representing individual animals at the indicated collection day. Resistance genes are identified at the bottom, treatment group and day of fecal collection on the left, and the animal numbers on the right. The color gradient key displays a linear scale of the percentage gene coverage as a measure of relative gene frequency.

In addition to beta-lactamases, additional resistance genes were affected by antibiotic exposure (Figure 7 and Supplemental Figure S2). Following amoxicillin/clavulanate alone treatment, increased frequency of some antibiotic resistance genes was observed, including those that encode multidrug efflux transporter system components that confer resistance to a broad range of antibiotics. Notably, several antibiotic resistance genes were lost following amoxicillin/clavulanate exposure, mainly genes conferring resistance to tetracycline and aminoglycosides. In all cases, SYN-007 attenuated changes to the gut resistome.

Results of the microbiome and resistome analyses demonstrate that oral administration of amoxicillin and amoxicillin/clavulanate caused alterations to gut microbiomes and resistomes. Amoxicillin/clavulanate exposure resulted in more dramatic shifts in gut microbiome and resistome composition compared to amoxicillin alone. Co-administration of SYN-007 with amoxicillin or amoxicillin/clavulanate attenuated gut microbiome and resistome changes.

## 4. Discussion

Antibiotic inactivation within the GI tract represents a novel strategy to preserve the gut microbiome from antibiotic-mediated damage. SYN-007, an oral delayed-release formulation of the beta-lactamase enzyme ribaxamase [23], was shown previously to protect the gut microbiome from damage caused by oral amoxicillin [15]. Here, SYN-007 functioned in the presence of the beta-lactamase inhibitor, clavulanate, to protect the gut microbiome from oral amoxicillin/clavulanate without interfering with oral amoxicillin systemic absorption.

Notably, ribaxamase, derived from a class A serine beta-lactamase, is sensitive to beta-lactamase inhibitors in vitro [23], but was capable of protecting the gut microbiome from antibiotic-mediated damage when delivered with amoxicillin/clavulanate in dogs. Similarly, the ribaxamase precursor, P1A beta-lactamase, was effective in the presence of beta-lactamase inhibitors in humans treated with IV piperacillin/tazobactam [24], and in dogs that received IV piperacillin/tazobactam, IV amoxicillin/sulbactam, and IV amoxicillin/clavulanate [24]. These paradoxical observations may be explained by assuming sufficiently high beta-lactamase concentrations within the GI tract to overcome inhibition by tazobactam, sulbactam, and clavulanate. Indeed, in a Phase 2a clinical study, ribaxamase concentrations > 1,000,000 ng/mL were measured in the GI tract of subjects, and maximum soluble concentrations of the beta-lactamase inhibitors sulbactam and tazobactam did not block ribaxamase activity at the high enzyme levels detected in human intestinal fluid [11]. These observations suggest that ribaxamase, and the delayed-release ribaxamase formulation SYN-007, may have utility in humans when used with beta-lactam/beta-lactamase inhibitor combinations.

While SYN-007 did not significantly alter amoxicillin systemic absorption when delivered with oral amoxicillin or amoxicillin/clavulanate, it is noteworthy that amoxicillin serum pharmacokinetic (PK) curves were indistinguishable with amoxicillin/clavulanate +/™ SYN-007 at day 6 (Figure 2D). In contrast, the day 6 amoxicillin + SYN-007 PK curve showed a more rapid decrease in amoxicillin serum levels at later time points compared to amoxicillin alone (Figure 2B). This occurrence was described previously when screening several SYN-007 delayed release ribaxamase formulation candidates [15]. A plausible explanation for this observation is that a minute amount of beta-lactamase released prematurely in the upper small intestine could degrade some amoxicillin prior to its systemic absorption, resulting in somewhat reduced antibiotic systemic levels, detectable only at later time points when antibiotic blood levels were low. However, if some low-level beta-lactamase leakage occurred in the presence of a beta-lactamase inhibitor, the enzyme would be neutralized rapidly before antibiotic degradation occurred and systemic antibiotic levels would not be affected. Indeed, the beta-lactamase inhibitor may be functioning as a fail-safe mechanism to counteract any inadvertent enzyme release from SYN-007 in the upper small intestine.

Despite the potential advantage of clavulanate in augmenting SYN-007 GI tract utility, amoxicillin/clavulanate was more disruptive to the gut microbiome than amoxicillin alone. While both amoxicillin and amoxicillin/clavulanate exposure resulted in changes in the gut microbiome, including significant reduction alpha diversity, principal coordinate analysis revealed that gut microbiome beta-diversity was altered more severely with amoxicillin/clavulanate (Figure 4A). This conclusion is supported by direct visualization of microbiome species composition via a stacked bar charts (Figure 5) where monodomination [22] with *E. coli, M. hypermegale,* or *B. vulgantus* was observed after amoxicillin/clavulanate exposure in four of the five treated animals. The increased abundance of these bacterial species was detected after amoxicillin exposure, but levels approaching monodomination were not reached.

These data may have implications regarding the clinical observation that amoxicillin/clavulanate therapy results in significantly increased incidence of antibiotic-associated diarrhea (AAD) compared to treatment with amoxicillin alone [18]. Clavulanate was assumed to be the causative agent as it increases intestinal motility in children [18], and amoxicillin/clavulanate formulations containing lower clavulanate ratios reduced AAD rates [18,25]. However, data from the current canine study revealed that amoxicillin/clavulanate causes more alterations in gut microbiota composition than amoxicillin alone, suggesting that microbiome damage is a mechanism responsible for AAD. Amoxicillin/clavulanate is known to modify gut microbiome composition in humans [26,27], including overgrowth of *Escherichia* species [27], and co-administration of the probiotic, *Saccharomyces boulardii,* to healthy subjects with amoxicillin/clavulanate limited microbiome damage and reduced AAD rates [27]. To date, clinical analyses that directly compare gut microbiome alterations resulting from exposure to amoxicillin, amoxicillin/clavulanate, and/or amoxicillin/clavulanate formulations with differing amoxicillin/clavulanate ratios have not been reported. However, accumulated data suggest that prevention of gut microbiome damage caused by amoxicillin/clavulanate may reduce AAD incidence.

Here, we demonstrated that SYN-007 protected the gut microbiome from damage caused by amoxicillin and amoxicillin/clavulanate. SYN-007 co-administered with amoxicillin or amoxicillin/clavulanate preserved alpha diversity and resulted in less alteration of microbiota composition compared to antibiotic alone cohorts. In animals that received SYN-007 + amoxicillin/clavulanate, gut microbiomes were protected from monodomination with *E. coli, M. hypermegale,* and *B. vulgantus* (Figure 5). However, some microbiome changes were observed in the presence of SYN-007. *F. mortiferum,* present at low levels in all pretreatment animals, increased in abundance following exposure to amoxicillin alone or amoxicillin/clavulanate + SYN-007, in two or three animals, respectively. *F. mortiferum*, an anaerobic Gram-negative bacteria that is a normal inhabitant of the oropharyngeal and GI tracts, was suggested to play a role in growth inhibition of enteropathogens [28].

In addition to microbiome alteration, oral amoxicillin and amoxicillin/clavulanate exposure caused resistome alterations, and SYN-007 attenuated these changes. Beta-lactamase genes, mainly those encoding class A TEM beta-lactamases, emerged following antibiotic exposure [29]. Most of these genes were observed in the amoxicillin/clavulanate alone cohort, following antibiotic exposure, with Dog 12 displaying the most beta-lactamase genes (Figure 6). Notably, Dog 12 was the animal that showed 92% monodomination with *E. coli,* suggesting that *E. coli* harbored these genes. While bacterial taxa possessing specific resistance genes cannot yet be determined with our metagenomics sequencing platform, these data are consistent with the fact that *blaTEM* genes are commonly found in Proteobacteria [29], and the emergence of beta-lactam resistant *E. coli* has been reported in dogs treated with oral amoxicillin [30] and amoxicillin/clavulanate [31]. Similarly, in humans, amoxicillin/clavulanate exposure resulted in enrichment for beta-lactamase genes in fecal resistomes, although the bacterial species harboring these genes were not evaluated [26].

Many of the beta-lactamase genes observed in this study encode extended spectrum beta-lactamases (ESBLs), enzymes capable of inactivating a broad range of beta-lactam antibiotics including penicillins and cephalosporins [29]. Perhaps even more worrisome is the emergence of beta-lactamases with resistance to the beta-lactamase inhibitors clavulanate and sulbactam [29]. These inhibitor-resistant TEM (IRT) beta-lactamases, originally discovered in *E. coli* clinical isolates, represent a group of enzymes distinct from the ESBLs [29]. Infections caused by bacteria harboring ESBLs and IRTs are steadily increasing and greatly complicate infection control efforts [29]. Here, genes encoding IRT beta-lactamases, TEM-83 and TEM-79 [29], were observed in 4/5 and 2/5 dogs, respectively, following amoxicillin/clavulanate exposure but were not detected in the presence of SYN-007. In addition, these IRT genes emerged in the resistomes of Dog 12 and Dog 15, the same animals that displayed monodominance with *E. coli.* It is notable that amoxicillin/clavulanate exposure resulted in the emergence of many more beta-lactamase genes, including ESBLs and IRTs, than did amoxicillin alone. Importantly, SYN-007 co-administered with amoxicillin/clavulanate, attenuated emergence of these genes. These data suggest that limiting the exposure of the gut microbiome, a reservoir of antibiotic resistance [6], to antibiotics with the use of beta-lactamases to inactivate antibiotics in the GI tract represents a viable approach to attenuate antibiotic resistance.

Other resistance genes were also affected by antibiotic exposure. Amoxicillin/clavulanate administration resulted in more pronounced changes to fecal resistomes than amoxicillin alone. An increased frequency of genes encoding multidrug efflux transporter system components that confer resistance to many antibiotic classes was observed with amoxicillin/clavulanate. Genes that displayed a decreased frequency with antibiotic exposure included genes conferring resistance to tetracycline and aminoglycosides. Co-administration of SYN-007 with amoxicillin and amoxicillin/clavulanate attenuated gut resistome changes. These observations suggest that alterations in the resistome were influenced by both direct selection of species resistant to beta-lactams, and by fluctuations in gut microbiota composition following antibiotic exposure. Changes in the frequency of a diverse set of antibiotic resistance genes following antibiotic exposure in dogs and pigs was reported previously [9,30,32,33].

## 5. Conclusions

Protection of the gut microbiome from damage caused by antibiotics is critical for maintenance of health, preservation of colonization resistance, and mitigation of antibiotic resistance emergence. Broad-spectrum antibiotics such as the beta-lactams are especially harmful. Here, the beta-lactam/beta-lactamase inhibitor combination amoxicillin/clavulanate was demonstrated to cause more damage to the gut microbiome than amoxicillin alone and resulted in the emergence of genes encoding ESBL and IRT beta-lactamases. SYN-007, a beta-lactamase formulated for delayed release following oral delivery with oral beta-lactam antibiotics, protected the gut microbiome of dogs from amoxicillin/clavulanate and reduced emergence of antibiotic resistance. The use of beta-lactamase enzymes to degrade antibiotics within the GI tract may represent a viable strategy for the protection of the gut microbiome and offers a pharmacologic stewardship approach to combat the emergence of antibiotic resistance.

## Supplementary Materials

The following are available online at https://www.mdpi.com/2076-2607/8/2/152/s1; Figure S1: Principal coordinate analyses of fecal microbiomes. Fecal microbiomes for each animal at each time point were subjected to principal coordinate analysis using Jaccard dissimilarity [21]. Amoxicillin alone pretreatment, pink, Amoxicillin alone post-treatment, red; Amoxicillin + SYN-007 pretreatment, gray, Amoxicillin + SYN-007 post-treatment, black; Amoxicillin/Clavulanate alone pretreatment, yellow, Amoxicillin/Clavulanate alone post-treatment, dark blue, Amoxicillin/Clavulanate + SYN-007 pretreatment, light blue, Amoxicillin/Clavulanate + SYN-007 post-treatment, green; Figure S2: Heatmap analysis of the frequency of antibiotic resistance genes in the dog fecal microbiomes. Fecal microbiome metagenomics data were analyzed for the presence of antibiotic resistance genes based on the percentage gene coverage as a measure of relative gene frequency in each sample. Each row represents an individual animal at the indicated time point. Antibiotic resistance genes are displayed at the bottom, treatment group and day of sample collection in the key on the right, and animal numbers on the right. The color gradient key displays a linear scale of the percent gene coverage as a measure of the relative gene frequency.
