# Supplementary material for: SYN-007, an Orally Administered Beta-Lactamase Enzyme, Protects the Gut Microbiome from Oral Amoxicillin/Clavulanate without Adversely Affecting Antibiotic Systemic Absorption in Dogs
Source: Microorganisms. 2020 Jan 22;8(2):152. doi: 10.3390/microorganisms8020152 (PMC7074739; doi:10.3390/microorganisms8020152)
Supplement: Supplementary file 1 [file microorganisms-08-00152-s001.pdf]

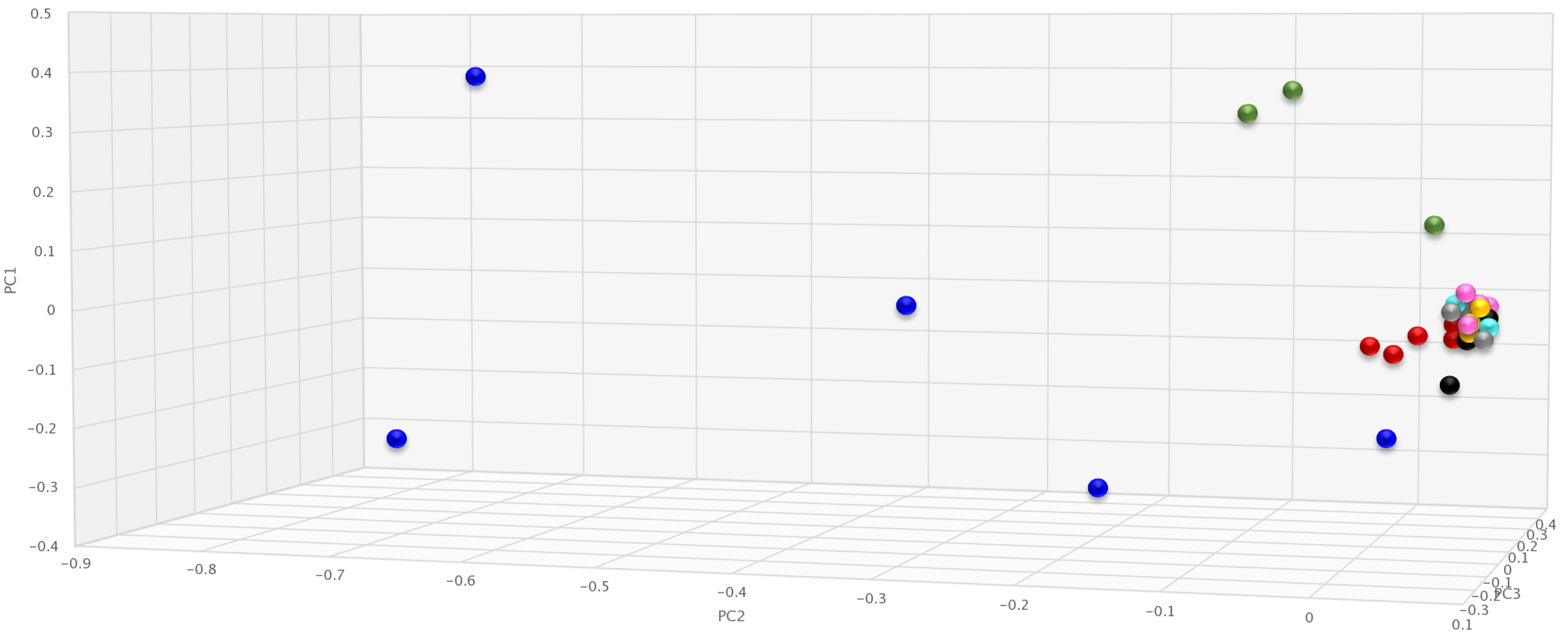

Supplemental Figure 1. Principal coordinate analyses of fecal microbiomes. Fecal microbiomes for each animal at each time point were subjected to principal coordinate analysis using Jaccard dissimilarity [21]. Amoxicillin alone pretreatment, pink, Amoxicillin alone post-treatment, red; Amoxicillin + SYN-007 pretreatment, gray, Amoxicillin + SYN-007 post-treatment, black; Amoxicillin/Clavulanate alone pretreatment, yellow, Amoxicillin/Clavulanate alone post-treatment, dark blue, Amoxicillin/Clavulanate + SYN-007 pretreatment, light blue, Amoxicillin/Clavulanate + SYN-007 post-treatment, green.

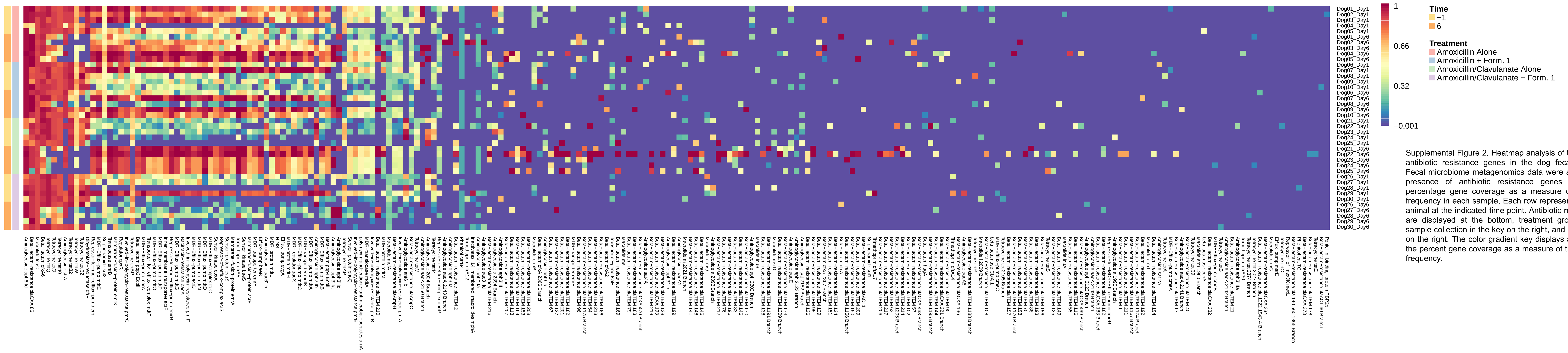

Supplemental Figure 2. Heatmap analysis of the frequency of antibiotic resistance genes in the dog fecal microbiomes. Fecal microbiome metagenomics data were analyzed for the presence of antibiotic resistance genes based on the percentage gene coverage as a measure of relative gene frequency in each sample. Each row represents an individual animal at the indicated time point. Antibiotic resistance genes are displayed at the bottom, treatment group and day of sample collection in the key on the right, and animal numbers on the right. The color gradient key displays a linear scale of the percent gene coverage as a measure of the relative gene frequency.
